# A biomechanical study of the medial row tightening on stability of single stitch suture-bridge construct

**DOI:** 10.1186/s13018-022-03180-8

**Published:** 2022-07-15

**Authors:** Sharon Abihssira, Pierre-Alban Bouche, Claire Cammas, Clément Thirache, Geoffroy Nourissat

**Affiliations:** Clinique Maussins-Nollet, Paris, France

**Keywords:** Suture-bridge, Biomechanics, Limb, Knot, Sutures, Tapes, Rotator cuff

## Abstract

**Purpose:**

To define the more stable knot tightening of a suture bridge when a single limb is preserved.

**Methods:**

Five different sutures were tested: No. 2 Ethibond (Ethicon), Hi-Fi (ConMed Linvatec), Sutblue (SBM), SingleFlat (SBM), Hi-Fi Ribbon (ConMed Linvatec). A Surgeon’s Knot was tied around a 30-mm circumference device, 6 times for each experiment. A single limb was kept to analyze failure modes of the knot. First step was to analyze which of pulling or sliding suture of the construct must be kept preventing failure of the knot. The cutting distance from the knot was evaluated at 1 mm and 4 mm with the suture loops pre-tensioned to 10 N and fixed to a second row after a 50 N tension load. The more stable construct was found: a single-pull load to 100 N and cyclic load (to 50 N for 30 cycles) experiments were conducted to evaluate the impact of cycling on knot loosening.

**Results:**

The more stable construct was obtained when the non-post limb was tensioned, and the post limb was cut at 4 mm (*p* < 0.01). Loop circumference increased after each experiment for all tested sutures, independently of the preserved limb and the cutting distance. Elongation was significant for all tested sutures in all groups. Knot failure mostly occurred by slippage, only with tapes.

**Conclusions:**

A suture-bridge construct with the non-post limb preserved and the post limb cut at a 4 mm distance from the knot provides with the best security. Sutures are safer than tapes in suture bridge.

## Introduction

Rotator cuff tears are mainly treated with arthroscopic methods after an initial medical management. Debridement and repair techniques are the most frequent treatments [1]. Among the repair techniques, suture bridge or transosseous equivalent is validated. It appears to be associated with a primary higher tendon healing rate and a reduced rate of retear compared to single-row repair [2, 3]. Different types of medial row configurations have been proposed. Suture bridge is different from speed bridge by the fact that the first row is tightened with knots, allowing compression and independent fixation at the medial row [4]. Double-mastress suture bridge combines a high fixation strength without mechanical failure [5]. This construct allows to remove one limb of each knot, leaving only one stich. The benefit of this technic is to decrease the volume of material sutures below the acromion and the size and/or number of lateral anchorages dividing by two lateral sutures. Knot configuration, loop security and suture materials have been evaluated in previous biomechanical studies based on single-row constructs. The impact on pulling on the remaining stiches, like done in suture bridge, has not been previously evaluated. Static surgeon’s knot with reversing half-hitches is associated with the best loop and knot security [6]. No study has measured the knot security relating to cutting distance from the knot or conserved suture limb (between the post and non-post limb) in mastress suture bridge. The purpose of this study was to define the more stable construct when a single limb is preserved in a suture bridge repair and evaluate the safe cutting distance from the knot. Additionally, we evaluate knot and loop security of this construct with five different types of suture material.

## Material and methods

**Fig. 1:**
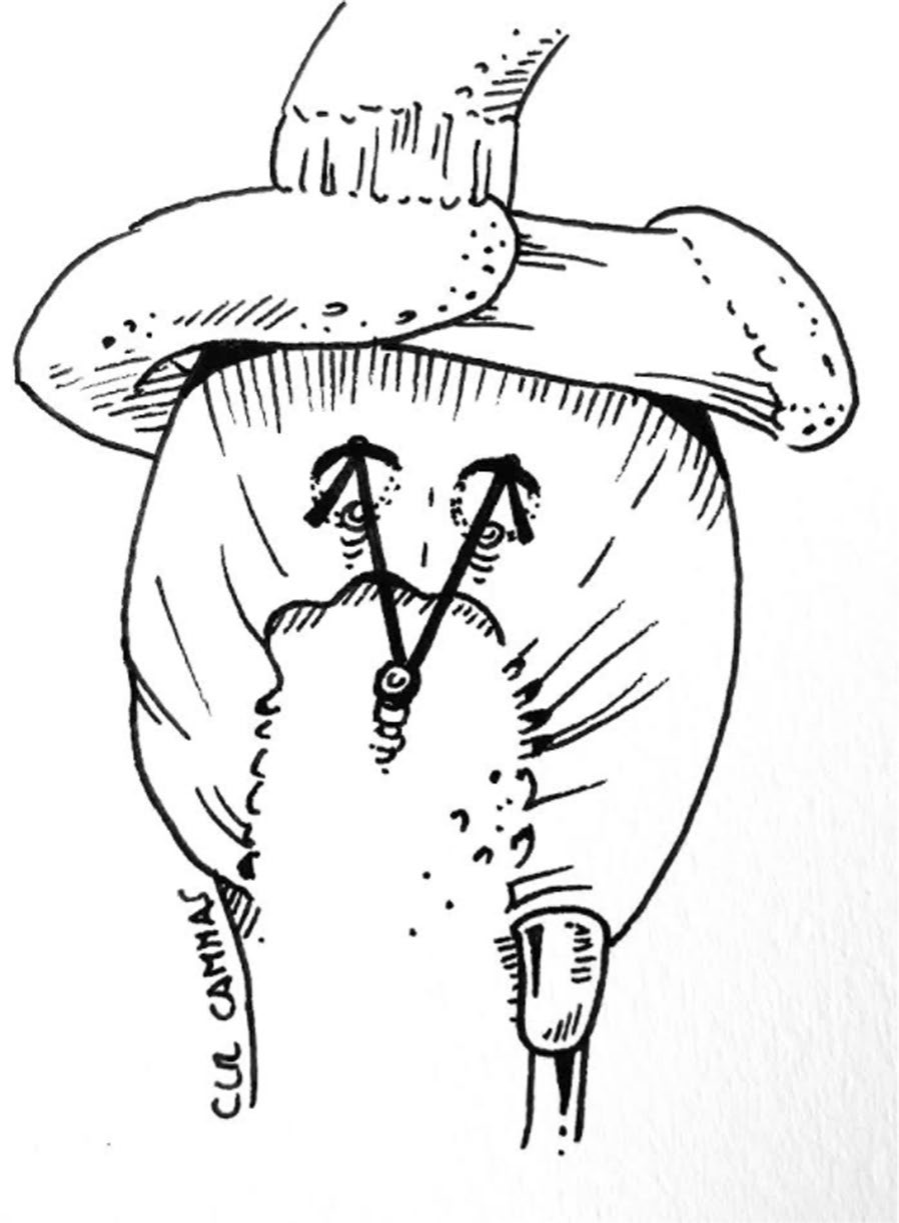
Views of the suture-bridge configuration tested

This biomechanical study was performed following a custom-made device based on a previous paper [6]. The evaluated construct is represented in Fig. 1. All knots were hand tied by a senior orthopedic surgery resident (C.T) on a custom-made support over two parallel metallic rods to create a 30 mm suture loop, replicating the optimal loop security in arthroscopic cuff repair [7, 8]. To recreate a standard arthroscopic procedure, knots were tied through an arthroscopic canula fixed to the support and using an arthroscopic knot pusher. This was done in a dry environment under direct visualization. A dynamometer (SF-100, Tanus^®^) was used to apply a constant load and realize the cyclic loading, up to 100 Newtons. For each experience, the traction was pulled along a longitudinal axis between the knot and the dynamometer.

**Fig. 2:**
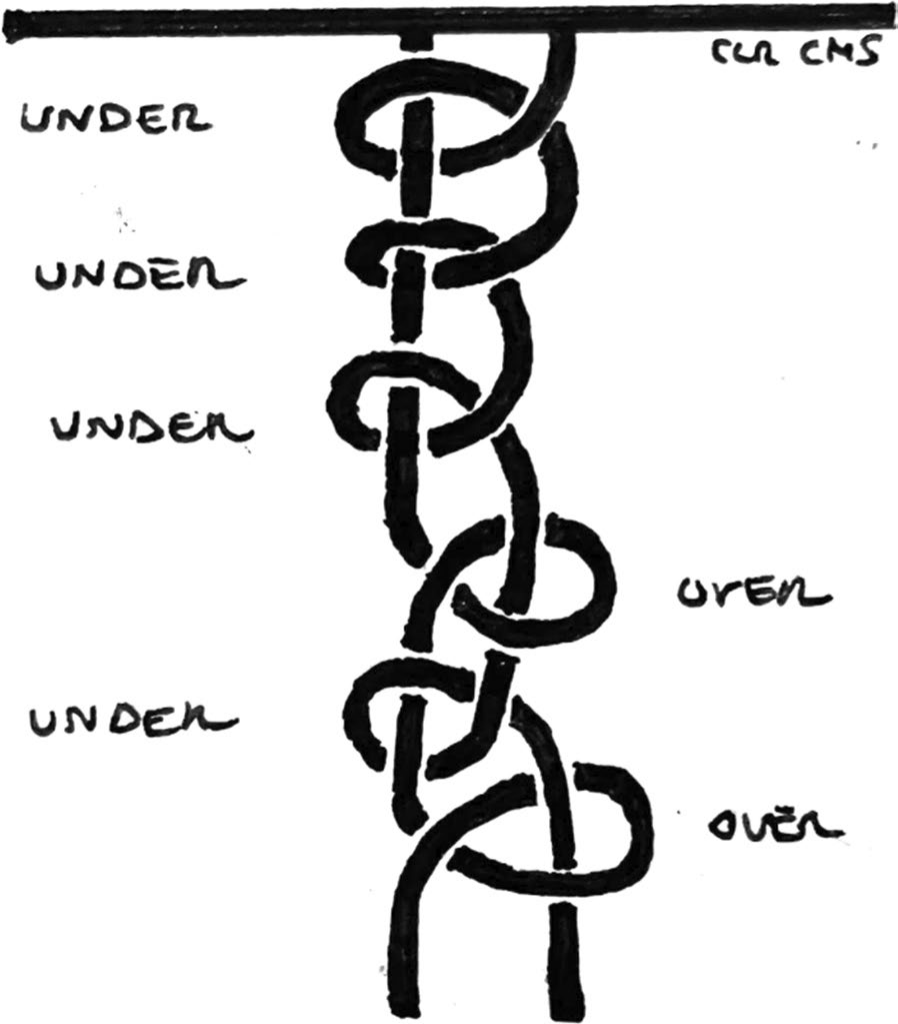
Static surgeon’s knot configuration

The Static Surgeon’s Knot was chosen for this study as it was the most commonly performed by the senior author (G.N). This knot comprised a stack of 3 half-hitches (base knot) followed by three consecutives half-hitches on alternating posts (Fig. 2).

We compared five different braided sutures and tapes: No. 2 Ethibond (Ethicon, Somerville, NJ), Hi-Fi (ConMed Linvatec, Largo, FL), Sutblue (SBM, Lourdes, France), SingleFlat (SBM, Lourdes, France), Hi-Fi Ribbon (ConMed Linvatec, Largo, FL). For each experiment, every single suture was used to tie the knot six times to be consistent with previous studies [6, 9].

The aim of the study was to know what is the more stable condition of the knot when cutting one stich of the suture before applying the second row.

Thus, we aimed to determine if there is a difference in knot loosening if we cut the post limb or the non-post limb and at which distance from the knot should the suture be cut. This loosening is commonly defined as the sliding of the suture. If it does not occur, measurement of the length of the loop circumference is an objective way to compare construct loosening.

We have successively done the four following experiments. In the first experiment, a Surgeon’s Knot was tied around the rods and a 10-N pre-load was applied on the post limb [10]. The non-post limb was cut at 1 mm (*n* = 6) and 4 mm (*n* = 6) with two distinct arthroscopic knot cutters (group A and B respectively). A tension load of 50 N was applied on the post limb for 10 s.

In the second experiment, we repeated the procedure with the post limb cut at 1 mm (*n* = 6) and 4 mm (*n* = 6) (groups C and D, respectively) and the tension load of 50 N applied on the non-post limb.

Those two experiments allowed us determining the limb to preserve and the safe cutting distance from the knot for each suture material tested.

Based on those results, in a third experiment, we tied the Surgeon’s Knot around the rods, cut the determined limb at the more stable length from the knot and fixed the other limb with a vice after a 50 N pre-load). We applied an opposed traction of 100 N to search for mechanical failure [11].

On the fourth experiment, we repeated the construct described above and performed a cyclic loading of 50 N during five seconds for 30 cycles to mimic chronic pulling strength as described in literature [10, 12].

For each experiment we took initial pictures, and videos during traction or cyclic loading to document mechanical failure. We reported loop circumference and knot slippage or breakage. We recorded the amount of suture elongation.

Statistical analysis was accomplished by use of *R* 3.5.0 software. Statistical significance was set for a *p* value < 0.05 and a power of 0.80. Comparison of continuous variables was done using the Kruskall–Wallis test.

## Results

### Experiments 1 and 2: loop and knot security with different limb in traction and short limb length

**Fig. 3:**
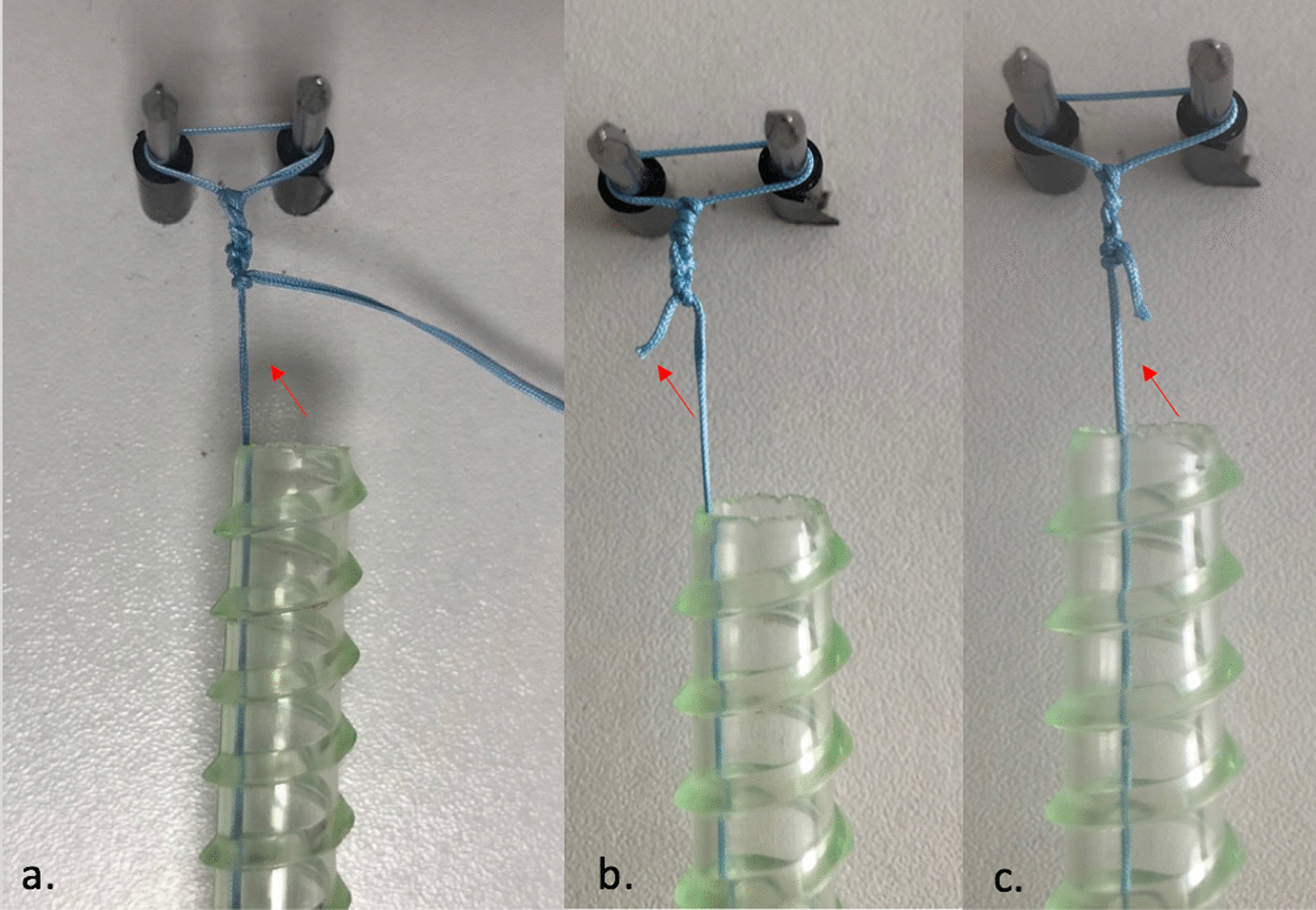
**a** Aspect of a Static surgeon’s knot before tension with tape (SingleFlat^®^) **b** Aspect of a stable construct in group D **c** Aspect of knot slippage in group A (knots tightened with Sutblue^®^). The red arrow indicates the post limb in each case

Those two experiments evaluated the stability of different double-mastress suture-bridge constructs with a single limb preserved as following: a non-post limb cut at 1 mm distance in group A or 4 mm in group B; a post limb cut at 1 mm distance in group C or 4 mm in group D (Fig. 3).

Table 1 includes the loop circumferences of each group. The loop circumference of the static surgeon’s knot tied with No. 2 Ethibond is, in all cases, the largest. There is a statistically significant difference between the tested braided sutures in all four groups for loop circumference and elongation (*p* < 0.01). Neither the preserved tensioned limb nor the cutting length altered the loop circumference when the groups were compared, independently of the suture type (*p* = 0.99). Suture elongation was minimal with Sutblue in all groups.

**Table 1:** Loop circumference in millimeters of static surgeon’s knot tied with various sutures types after a 50 N tension load with different constructs (Group A: non-post limb cut at 1 mm; Group B: non-post limb cut at 4 mm; Group C: post limb cut at 1 mm; Group D: non-post limb cut at 4 mm)

Group	Ethibond 2	Hi-Fi	Sutblue	Hi-Fi Ribbon	SingleFlat 2	*p* value
A	33 [33;34.5]	32 [32;32.75]	30 [30;30.75]	31 [31;32.5]	30 [29.25;30]	** < 0.01**
B	33 [33;33]	32 [30.88;32.75]	31 [30.25;31]	31 [31;31.75]	30 [29.25;30]	** < 0.01**
C	33 [33;33]	32.5 [32;33.75]	30 [30;30]	31 [31;31]	29 [28;30]	** < 0.01**
D	33 [33;33]	32.5 [32;33.75]	30 [30;30]	31 [31;31]	29 [28;30]	** < 0.01**

Failure mechanism mainly occurred by knot slippage (Fig. 3). It was the lowest in group D, when the non-post limb was tensioned and the post limb was cut at 4 mm, compared to the other groups (Table 2, *p* < 0.01). All kind of suture slipped when the tensioned limb was the post limb (group A and B). Knot breakage was observed only once with No. 2 Ethibond. There was no statistical difference between the sutures in group A, B or C (*p* > 0.05). In group D, we noted knot slippage only with SingleFlat2 (*p* = 0.03).

**Table 2:** Failure mode in each tested construct according to suture type (Group A: non-post limb cut at 1 mm; Group B: non-post limb cut at 4 mm; Group C: post limb cut at 1 mm; Group D: non-post limb cut at 4 mm)

	Group A	Group B	Group C	Group D
No 2. Ethibond	83% Slipped, *n* = 5	50% Slipped, *n* = 3 17% Broke, *n* = 1	17% Slipped, *n* = 1	0
Hi-Fi	83% Slipped, *n* = 5	100% Slipped, *n* = 6	0	0
Sutblue	67% Slipped, *n* = 4	67% Slipped, *n* = 4	17% Slipped, *n* = 1	0
Hi-àlFi ribbon	100% Slipped, *n* = 6	83% Slipped, *n* = 5	17% Slipped, *n* = 1	0
SingleFlat 2	83% Slipped, *n* = 5	67% Slipped, *n* = 4	33% Slipped, *n* = 2	50% Slipped, *n* = *3*

### Experiment 3: loop and knot security after a 100 N single-pull load

The loop circumference was significantly higher for all sutures after a 100 N single pull load on a suture-bridge construct (*p* < 0.01). There was a significant amount of elongation for all the tested sutures (*p* < 0.0001). Elongation of the loop was minimal with Sutblue (0,05 ± 0,028) followed by Hi-Fi (0,1 ± 0,04), SingleFlat2 (0,1 ± 0,02) and No. 2 Ethibond (0,22 ± 0,03). It was the largest with Hi-Fi Ribbon (0,23 ± 0,03).

Failure occurred only by knot slippage and only with tapes (Table 3). We found no significant difference between the suture type (*p* = 0,47). Knot slippage was observed during the pre-tensioned step at 50 N when the post limb was fixed with the vice.

**Table 3:** Failure mode after a 100-N single-pull load

Suture	*n*	Knot slippage	Knot breakage
Ethibond 2	6	0	0
Hi-Fi	6	0	0
Sutblue	6	0	0
Hi-Fi ribbon	6	17%, *n* = *1*	0
SingleFlat 2	6	33%, *n* = *2*	0

### Experiment 4: loop and knot security after cyclic loading

Loop circumference and elongation were significantly higher for all sutures after cyclic loading (*p* < 0.01). Elongation of the loop was minimal with Sutblue (0,03 ± 0,02) followed by Hi-Fi (0,09 ± 0,05), SingleFlat2 (0,14 ± 0,01) and No. 2 Ethibond (0,15 ± 0,03). It was the largest with Hi-Fi Ribbon (0,17 ± 0,04).

No knot breakage was observed. Knot slippage concerned six out of the thirty knots tested. We found a significant difference between the suture types, and tapes were the less secure material (*p* = 0.043, Table 4).

**Table 4:** Failure mode after cyclic loading

Suture	*n*	Knot slippage	Knot breakage
Ethibond 2	6	17%, *n* = *1*	0
Hi-Fi	6	0	0
Sutblue	6	0	0
Hi-Fi ribbon	6	17%, *n* = *1*	0
SingleFlat 2	6	67%, *n* = *4*	0

## Discussion

To our knowledge, this study is the first to evaluate the mechanical properties of this specific suture-bridge construct and the safety of the cutting distance from the knot. A suture-bridge construct with the non-post limb preserved and the post limb cut at a 4 mm distance from the knot provides with the best security. Tapes tested in our study should not be recommended for this type of construct.

**Fig. 4:**
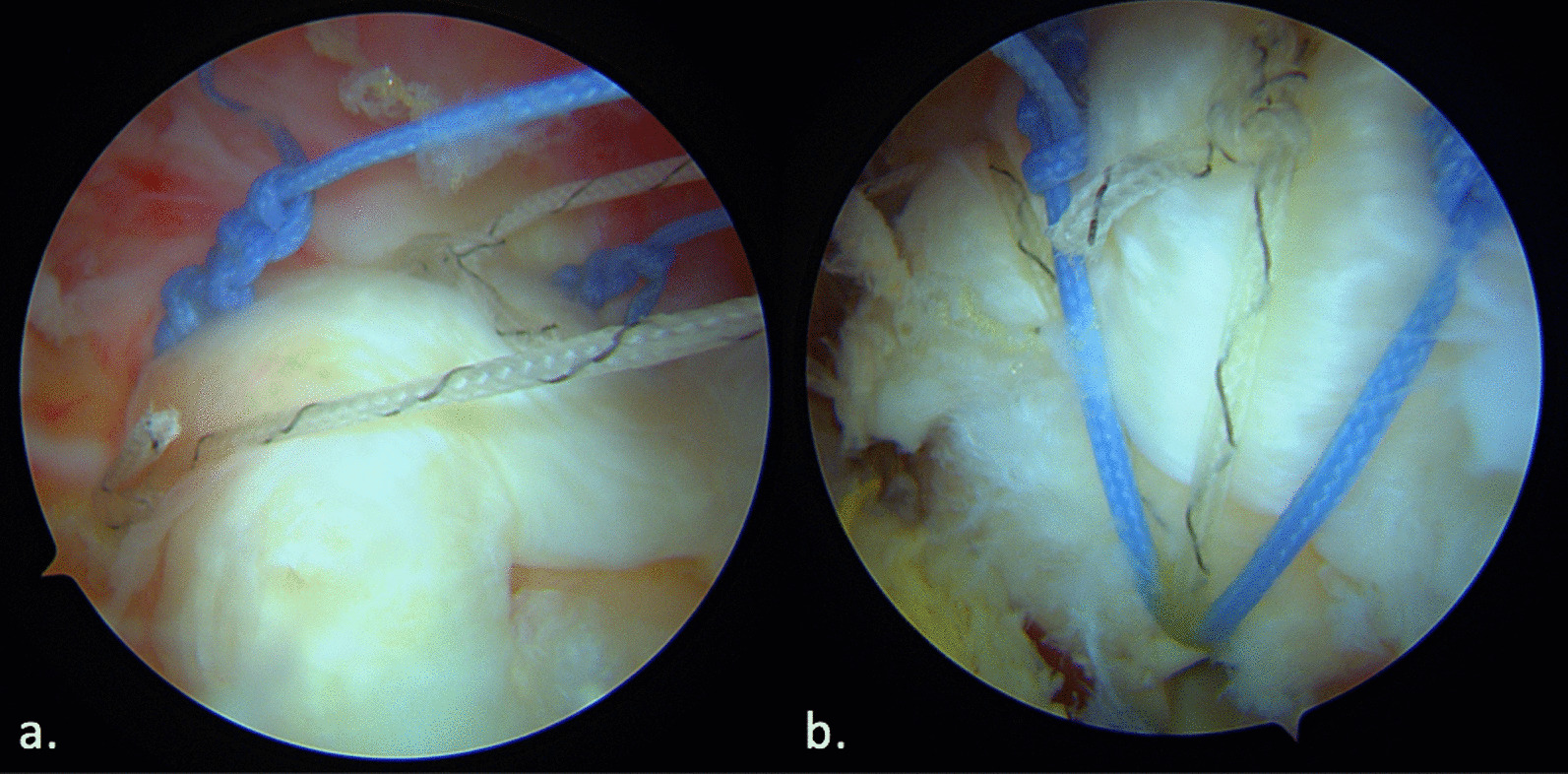
Suture-bridge equivalent construct **a** First row tightened with a Static surgeon’s knot using a Hi-Fi suture before applying the second row, **b** Final construct of suture bridge with single lateral stich configuration

Healing of the cuff is the main objective of shoulder cuff repair. Failed repair is correlated with poor outcomes and risk of progressive osteoarthritis. Several causes of failure are well identified; among them early failure or loosening of the suture material should be correlated to non-healing [13, 14]. Suture bridge has been developed to stabilize the first row of fixation and increase the contact between the bone of the tendon. Clinical results confirm its benefit, but increasing the number of lateral stiches increases the volume of material in the subacromial space and the size and/or number of anchors used for the lateral row. Removing one suture can decrease those risks but should not compromise stability (Fig. 4).

Numerous publications have focused on knot and loop security in a single-row construct with different types of knot tested. Our results are consistent with previous studies that demonstrate the best security with static surgeon’s knot and the importance of reversed half-hitches to secure the knot [6, 15]. They highlighted that the construct itself matters in knot security in a double-row configuration. When tension was applied on the post limb, it was associated with elongation and slippage of the last half-hitches. On the contrary, applying tension on the non-post limb allowed for a stable construct with less knot failure. It is also safer to cut the knot at a 4 mm distance than 1 mm distance.

To ensure the best loop security, we customized a stable mechanical model where the rods were separated to create a 30-mm suture loop as reported in literature [8]. The preserved limb and the cutting distance from the knot did not interfere with loop elongation (*p* = 0.99). Loop circumference increased with single-pull load and cyclic loading for all kind of sutures tested (*p* < 0.01).

We found that knot failure was limited when the non-post limb is preserved, and the post limb cut at a 4 mm distance from the knot. It occurred mainly with tapes and by slippage. The results are similar whether it is a single-pull load or a cyclic load experiment. Previous studies have demonstrated the advantages of using tapes rather than sutures in rotator cuff repair: higher load-to-failure, higher stiffness, and higher contact pressure between the bone and the tendon [16]. Despite their biomechanical properties, Liu et al. observed that tapes do not influence the retear rate [17]. Additionally, tapes have not been evaluated considering knot security. Considering the existing literature and our results, we do not recommend using tapes for double-row configuration constructs involving knots tying.

The strengths of this study are that the knots were all tied by a single surgeon, with the use of arthroscopic instrument and a standardized loop size, under same load device, following previous validated protocols [6–8].

The limitations are the variation in the tension applied to each knot as it was done by a surgeon and not mechanically by a machine. Even if the load applied was consequent, we could not study the load-to-failure with the dynamometer at our disposition. Experiments were done in a dry environment, and our results might differ when sutures are soaked in a biological media reproducing human body conditions. Rousseau et al. have shown that body fluid immersion influence knot slippage and differences exists between UHMWPE sutures [11]. This study confirmed that UHMWPE sutures were safer than No 2. Ethibond and tapes in this construct. Moreover, it has been demonstrated that polyester sutures are associated with higher rates of biological markers’ expression related to rotator cuff healing compared to Ethibond suture [18]. Biological and mechanical finding are actually in favor of UHMWPE sutures. In the future, it would be interesting to confirm our result in a biological environment. Statistical difference between suture material is not available as it would have come from multiple tests with no statistical power.

Additional studies could provide with knot and loop security in a wet environment and help differentiate more precisely sutures and tapes in this suture-bridge construct.

## Conclusion

A suture-bridge construct with the non-post limb preserved and the post limb cut at a 4 mm distance from the knot provides with the best security. Sutures we tested in our biomechanical study are safer than tapes in this double-row suture-bridge single stich configuration.

## Data Availability

This is a biomechanical study that was not done on human or animal subjects. All the data and material can be transferred if needed.
